# Nature of the Interaction of Alpha-D-Mannose and Escherichia coli Bacteria, and Implications for its Regulatory Classification. A Delphi Panel European Consensus Based on Chemistry and Legal Evidence

**DOI:** 10.1007/s43441-023-00548-8

**Published:** 2023-08-14

**Authors:** Francesco Scaglione, Paola Minghetti, Francesco Ambrosio, Beat Ernst, Vincenzo Ficarra, Marco Gobbi, Kurt Naber, Huub Schellekens

**Affiliations:** 1 Clinical Pharmacology and Toxicology Unit -GOM Niguarda, GOM Niguarda, Piazza Ospedale Maggiore 3, 20162 Milan, Italy; 2 Department of Pharmaceutical Sciences, University of Milan, Via Mangiagalli 25, 20133 Milan, Italy; 3 Bureau Veritas Group, Via Santa Brigida, 39, 80133 Napoli, Italy; 4 Group Molecular Pharmacy Pharmacenter, University of Basel, Klingelbergstrasse 50, 4056 Basel, Switzerland; 5 Department of Human and Pediatric Pathology “Gaetano Barresi”, Urologic Section, University of Messina, Piazza Pugliatti, 1, Messina, Italy; 6 Laboratory of Pharmacodynamics and Pharmacokinetics, Istituto Di Ricerche Farmacologiche Mario Negri IRCCS, Via Mario Negri, 2, 20156 Milan, MI Italy; 7 Department of Urology, Technical University of Munich, Munich, Germany; 8 Department of Urology, Technical University of Munich, Karl-Bickleder Str. 44C, 94315 Straubing, Germany; 9 Faculty of Sciences, Utrecht University, PO Box 80125, 3508 TC Utrecht, The Netherlands

**Keywords:** D-mannose, Urinary tract infection, FimH adhesin, Product classification, Delphi consensus

## Abstract

The nature of alpha-D-mannose—natural aldohexose sugar, C-2 glucose epimer, whose intended use is for preventing urinary tract infections—in the interaction with *E. coli* is addressed in order to drive the issue of its regulatory classification as a medicinal product or medical device. PRISMA systematic review approach was applied; Delphi Panel method was used to target consensus on statements retrieved from evidence. Based on regulatory definitions and research evidence, the mechanism of D-mannose does not involve a metabolic or immunological action while there is uncertainty regarding the pharmacological action. Specific interaction between the product and the bacteria within the body occurs, but its nature is inert: it does not induce a direct response activating or inhibiting body processes. Moreover, the action of D-mannose takes place, even if inside the bladder, outside the epithelium on bacteria that have not yet invaded the urothelial tissue. Therefore, its mechanism of action is not directed to host structures but to structures (bacteria) external to the host's tissues. On the basis of current regulation, the uncertainty as regard a pharmacological action of alpha-D-mannose makes possible its medical device classification: new regulations and legal judgments can add further considerations. From a pharmacological perspective, research is driven versus synthetic mannosides: no further considerations are expected on alpha-D-mannose.

**Supplementary Information:**

The online version contains supplementary material available at 10.1007/s43441-023-00548-8.

## Introduction

Urinary tract infection (UTI) is an infection of the urinary system that could affect the kidneys, the ureters, the bladder and the urethra. The pathogen that could cause UTIs in the urogenital tract and the bladder is *Escherichia coli (E. coli*) in approximately 53% of cases. Many people develop a single episode in their life (50% of them are female), and approximately 15% to 25% of adults and children suffer from chronic symptomatic UTIs, namely recurrent, persistent, re-infected or relapsed UTIs. Compared to men, women have a higher likelihood of developing a urinary tract infection (UTI). At present, available remedies and interventions for UTI include administering antibiotics, methenamine hippurate salts, topical estrogens, urine alkalisers, dietary supplements, and implementing modifications in lifestyle and behavior. In clinical practice, many patients do not respond to standard antibiotic treatments producing important patient burden and high cost to healthcare systems [1]. Escalating bacterial resistance to traditional antibiotics and limited efforts in developing new antibiotics require the identification of novel therapies.

The concept of “disarming” bacteria, rather than outright killing them, was first proposed in the 1980s. This approach has since driven extensive research in both structural biology and clinical settings, in contrast to other small molecule strategies that aim to prevent bacterial binding to urothelial cells [2, 3].

In UTI prevention, D-mannose utilized for N-glycosylation and glycerophospholipid anchor synthesis is derived from the enzymatic stereospecific interconversion of glucose rather than dietary intake various healthcare products in the European Union and the United States, including food supplements, contain alpha-D-mannose [4]. It was introduced to the European market in 2015 as a Class IIa medical device. The Borderline and Classification Medical Devices Expert Group identified D-mannose as a borderline product in their 2019 manual, excluding it from their list [5].

The criterion for establishing the regulatory framework in which a product may fall starts from the definition and identification of the mechanism by which it performs its main action [6]. A very relevant documentation supporting the discussion is the Guidance on Borderline Between Medical Devices and Medicinal Products Under MDR (MDCG 2022–5), in which the definition of medical device derives from the Art. 2 of the 2017/745 MDR regulation [7]. By this definition, for medical devices, the main action for intended use must not act in or on the human body by pharmacological, immunological or metabolic means, although it can be supported by one of these. However, the definitions of pharmacological, immunological or metabolic action are not always unique: distinction between mechanism of action and related actions in the organism could be unclear. The quality and nature of mechanism of action and intended use can be helpful aspects [6, 8].

The expert panel’s objective was to examine the role of alpha-D-mannose as a medicinal product or medical device in its interaction with *E. coli* bacteria, and their systematic review methodology adds further value compared to previous publications [4].

**Figure 1:**
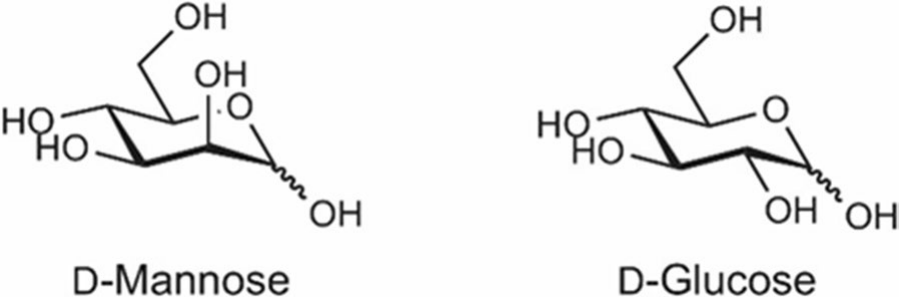
Chemical Structure of D-Mannose Compared to D-Glucose.

BOX 1: D-Mannose—Chemistry and Physiology We explore the chemical and physiological properties of D-Mannose, a naturally occurring aldohexose sugar that is a C-2 epimer of glucose. This means that it differs from glucose in only one of its chiral centers, specifically the carbon atom 2. (Fig. 1) [4]. D-mannose is one of the nine monosaccharides (D-glucose, D-galactose, D-mannose, D-xylose, L-fucose, D-glucuronic acid, N-acetyl-D-glucosamine, N-acetyl-D-galactosamine, and N-acetylneuraminic acid) commonly found in animal glycans and in vertebrate glycoconjugates [2].

D-mannose, which has a physiological blood concentration less than one-fiftieth of that of glucose, is physiologically present in the human body. D-mannose, a crucial molecule involved in glycoprotein synthesis, can be synthesized primarily from glucose or derived from endogenous lycoconjugates. Upon catabolism via glycolysis, it serves as a source of energy or can be incorporated into glycans.

D-mannose occurs naturally in many plants and fruits, especially cranberries, in relatively small amounts. However, the bioavailability of mannose for glycan synthesis in these dietary sources is poor, therefore, dietary mannose is not considered as a significant source of D-mannose for humans.

D-mannose has gained significant attention for its ability to prevent urinary tract infection, both as a standalone supplement and when combined with cranberry extract or probiotics. Despite its simplicity as a sugar, D-mannose remains unmetabolized within the human body [9–13], thereby highlighting its potential as an effective agent for preventing urinary tract infectionPharmacokinetic studies have shown that at least 90% of ingested D-mannose is efficiently absorbed in the upper intestine, and rapidly excreted from the bloodstream. Its plasma half-time ranges from 30 min to some hours. Within 30–60 min, a substantial portion of D-mannose is excreted unaltered in the urine, while the remaining amount is expelled over the subsequent 8 h [9]. Roughly 20–35% of the dose of excess D-mannose infiltrates the urine from the bloodstream within an hour, presenting an opportunity to engage with the mannose-sensitive structures of uropathogenic E. coli (UPEC) and mitigate their harmful effects [2].

Notably, this process does not result in a significant elevation of glucose levels and D-mannose only appears at trace levels in tissues.

## Materials and Methods

### Delphi Panel

The consensus was structured according to the modified Delphi panel method [14].

The expert group was formed representing the key expertise with a specific interest in the topic: key opinion leaders were covering different fields to include in the discussion the point of view of the physicians preventing UTIs in their everyday life (urologists) and of the pharmacologists/biotechnologists for their deep knowledge of the different interactions leading to therapeutic action. The point of view of professionals with thorough expertise in the regulatory process of medicinal products and medical devices was also integrated. The panel consisted of representatives from various European nations, and their collective median h-index ranged from 38 to 74 with a value of 48 [range: 38–74].

A Likert scale, graded from 1 to 7, was employed to gauge opinions, with 1 signifying no concurrence and 7 indicating complete agreement. Agreement was defined by a threshold of 6, whereas a score of 5 was considered indicative of indecision [15].

### Prisma Research

Prisma 2020 expanded checklist and flow diagram were used as appropriate to systematic reviews of studies that evaluate the effects of health interventions [16]. All studies (in vitro and in vivo) explicitly related to the mechanism of action of D-mannose in the binding of *E. coli* preventing UTI were included. All abstracts were included. All other articles considered relevant by the panel, as far as the regulatory and pharmacological point of view, guidelines and directives, including papers from references were manually retrieved. Research papers that lacked abstracts, studies that investigated the clinical efficacy of D-mannose, studies that focused on pathogens other than *E. coli*, and abstracts written in languages that necessitated translation, such as Russian, Bulgarian, and Japanese, were omitted from consideration.

The PubMed database was searched out on 10th October 2022. MESH research was implemented (“Urinary Tract Infections/drug therapy”[Mesh] OR “Urinary Tract Infections/etiology”[Mesh] OR “Urinary Tract Infections/immunology”[Mesh] OR “Urinary Tract Infections/metabolism”[Mesh] OR ”Urinary Tract Infections/microbiology”[Mesh] OR “Urinary Tract Infections/physiology”[Mesh] OR “Urinary Tract Infections/physiopathology”[Mesh] OR “Urinary Tract Infections/prevention and control”[Mesh] OR ”Urinary Tract Infections/therapy”[Mesh])) AND “Mannose”[Mesh]). A search without MESH was also implemented ((D-mannose [Title/Abstract]) AND (urinary tract infection*[Title/Abstract]). No limits were set on timeframe and languages.

Articles were manually retrieved. Three external reviewers (SL, LP, SG) screened each record and each report retrieved (title/abstract). Multiple reviewers (FS, MG, BE) worked independently at each stage of screening and an email process was used to resolve disagreements between screeners.

Data collection process was manually performed by three reviewers (SL, SG, LP) who independently worked. Synthesis methods included all the included studies which were qualitatively tabulated according to the study type (in vitro, in vivo, review) and main results relevant to describe the nature of the interaction between D-mannose and *E. coli*. Since the review does not regard clinical outcomes assessment, no risk of bias and no aggregated data could be estimated and synthesis of qualitative has been consulted [17, 18].

### Statements Drafting

**Figure 2:**
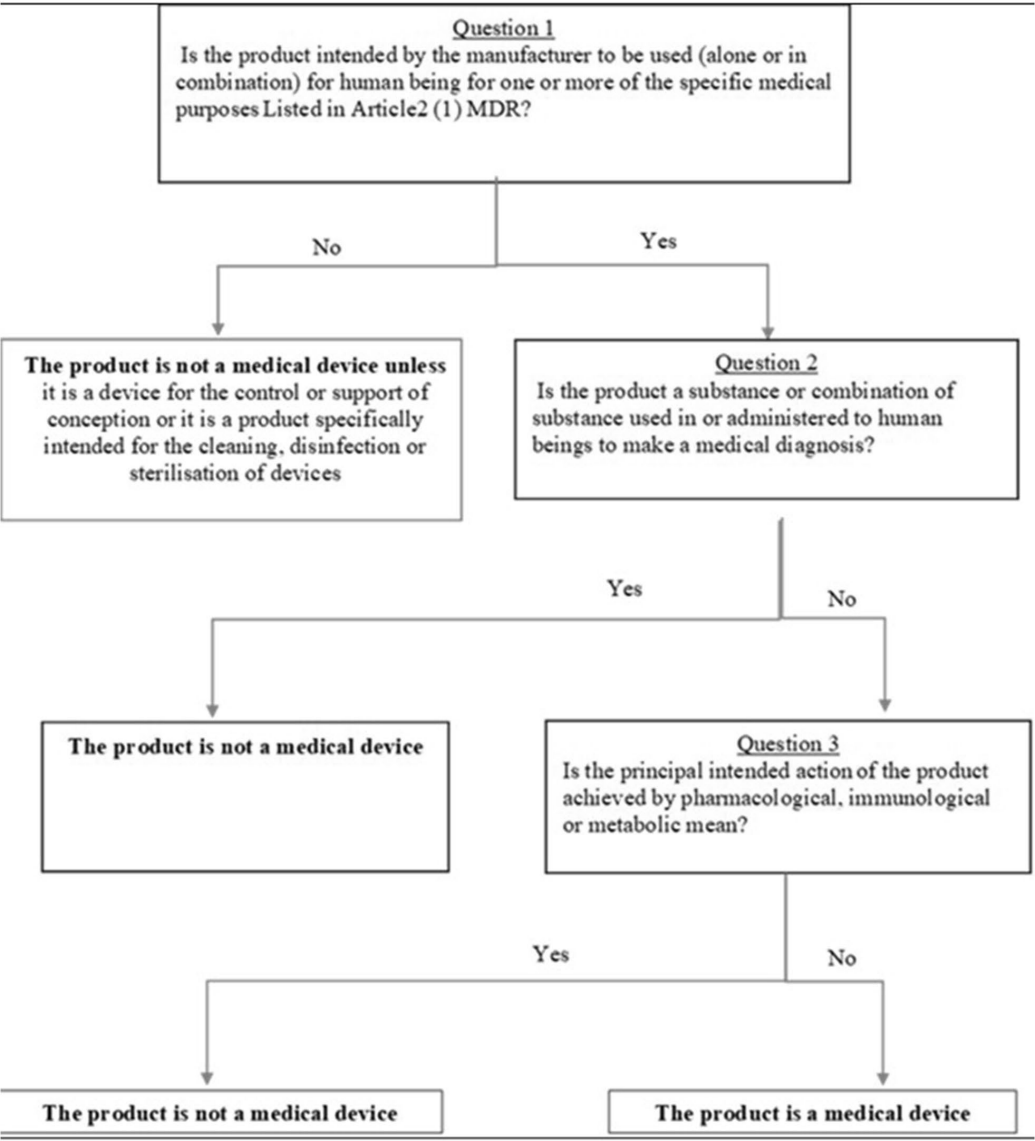
Flowchart for Determining if a Product Fulfils the Definition of a Medical Device (MDCG 2002–5) [7].

The research question has been discussed based on the Medical Device Coordination Group (MDCG) 2022–5 guidelines (Fig. 2) [7] and art. 2 of the regulation 2017/745 (MDR) [19]^1^ and available evidence on the mechanism of action of D-mannose for UTI prevention*.*

## Results

### Prisma Results

**Figure 3:**
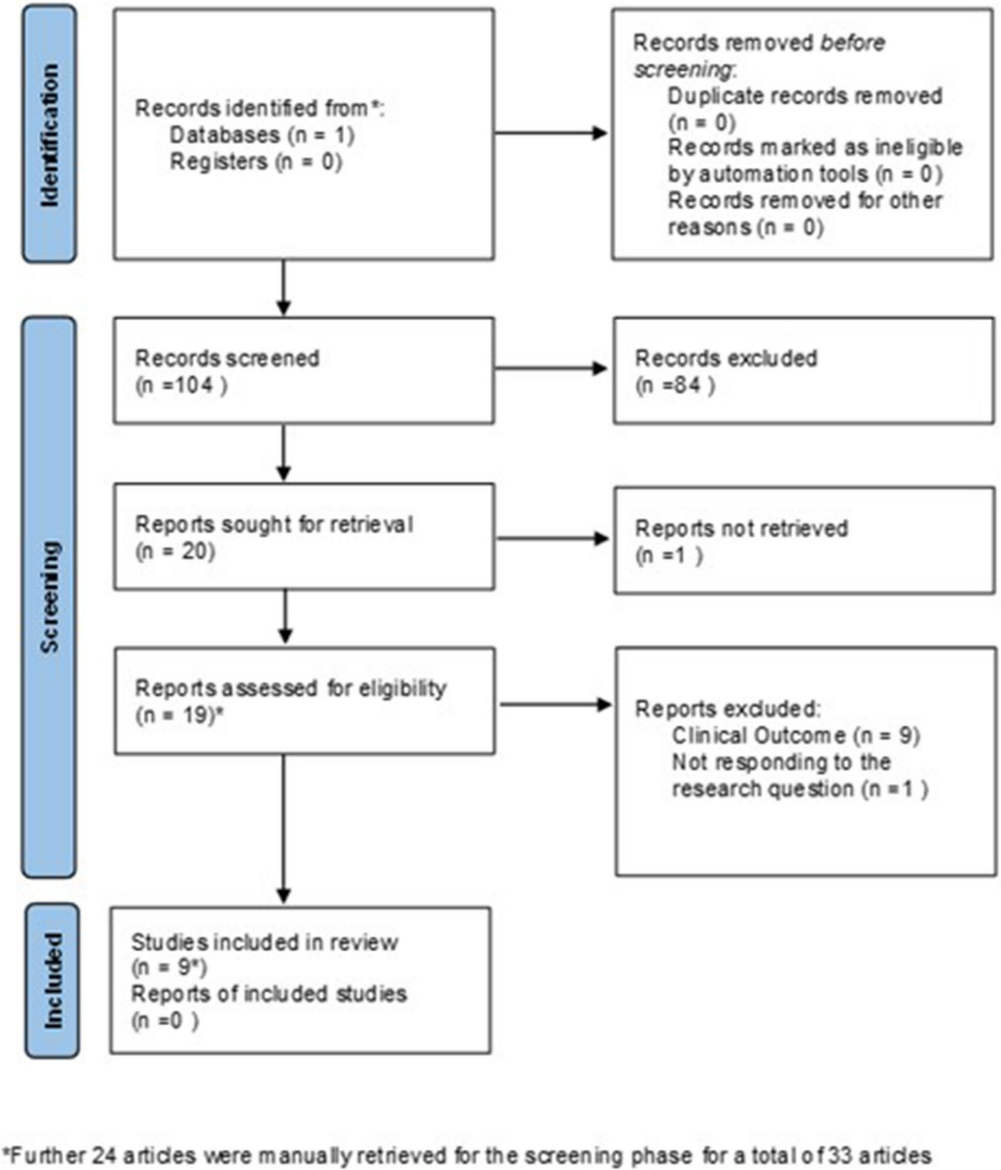
PRISMA Flowchart of the Selection Process of Articles that Fulfilled the Criteria.

A total of 33 articles were retrieved (Fig. 3). Studies characteristics, results of individual studies and synthesis are reported in the supplement materials.

### Delphi Panel Results

Following a kick-off virtual meeting, a series of 17 statements were sent to the panelists via email. During Round I, experts were allowed to provide comments and suggest rephrasing or additional items. During Round 2, the statements that did not meet consensus after the first round were rephrased according to panellists’ comments: one statement was cancelled. During a final teleconference (29th November 2022), statements which had not reached a consensus after Round II were discussed and reviewed for the merit of inclusion and statements that had already reached a consensus were reviewed, discussed and validated: 3 statements were merged. In the end, 13 statements were drafted, 4 of which regarding definitions and the remaining specifically regarding alfa-D-mannose. Statements are reported in Table 1.

**Table 1:** Statements Regarding Definitions and Alpha-D-Mannose Interactions: Related Delphi Panel Votes.

Statement on Definitions			
	Statement	Delphi Panel Round I Voting (Average)	Delphi Panel Round II Voting (Average)
N#1	Regulatory perspective regarding Medicinal Device According to the panel, the pharmacological definition of drug is included in the regulatory perspective on medicinal product: a) any product or combination of products presented as having properties for treating or preventing disease in human beings; b) any product or combination of products which may be used in or administered to human beings either with a view to restoring, correcting or modifying physiological functions by exerting a pharmacological, immunological or metabolic action, or to making a medical diagnosis Therefore, the MDGC definition insists on mechanism of action by which the medicinal products produce its activity [7, 20] Regulatory perspective regarding Medical Device The MDCG 2022–5 guidelines adds a relevant aspect: it indicates that medical device does not achieve its principal intended action by pharmacological, immunological or metabolic means, in or on the human body, but which may be assisted in its function by such means	6	8
N#2	Pharmacological action According to the MDCG 2022–5, the pharmacological action is an interaction at a molecular level between a product or its metabolites and a constituent of the human body which results in initiation, enhancement, reduction or blockade of physiological functions or pathological processes Examples of constituents of the human body may include, among others: cells and their constituents (cell membranes, intracellular structures, RNA, DNA, proteins, e.g., membrane proteins, enzymes), components of extracellular matrix, components of blood and components of body fluids Moreover, the EU jurisprudence clarified that in order for a product to be regarded as having a ‘pharmacological action’ an interaction with any cellular component present in the user’s body (e.g., bacteria, viruses, or parasites) is enough, if this influences positively the physiological functions of the human body Although not a completely reliable criterion, the presence of a dose–response correlation is indicative of a pharmacological effect [7, 21–24]	7	8
N#3	Immunological action According to the MDCG 2022-5The immunological action is initiated by a product or its metabolites on the human body and mediated or exerted (i.e., stimulation, modulation, blocking, replacement) by cells or molecules involved in the functioning of the immune system (e.g., lymphocytes, toll-like receptors, complement factors, cytokines, antibodies) [7]	7	8
N#4	Metabolic action According to the MDCG 2002–5, the metabolic action involves an alteration, including stopping, starting or changing the rate, extent or nature of a biochemical process, whether physiological or pathological, participating in, and available for, function of the human body [7]	8	8
Statements on D-mannose			
N#5	D-mannose is indicated to prevent the recurrence of cystitis and other uncomplicated infections of the lower urinary tract uncomplicated Urinary Tract Infections (uncomplicated UTI)	7	7
N#6	D-mannose is not indicated to prevent the acute episode of recurrence of cystitis and other uncomplicated infections of the lower Urinary Tract (uncomplicated UTI)	8	8
N#7	D-mannose binds the bacterial FimH adhesin and prevents interaction with mannsylated proteins or lipids on urothelial cells The interaction of the uropathogenic *E. coli* (UPEC) with mannosylated proteins on the bladder epithelium is the main mechanism to initiate the infection (Box 2). This interaction occurs via the FimH adhesin located at the tip of the type I fimbria of *E. coli*, which is the virulence factor in UTI pathogenesis (See box 1 for details) [23–42] Accordingly, FimH was identified as a therapeutic target in the late 1980s, a substantial body of research has been generated focusing on the development of FimH-targeting mannose-based anti-adhesion therapies The rationale to the use of D-mannose in UTIs prophylaxis is therefore based on its competitive inhibition of bacterial adherence to mannosylated urothelial cells [12, 43, 44]. In other words, FimH on *E. coli* can no longer bind to urothelial cells, preventing the adhesion abilities of the bacterium. The inhibition of the adhesion step thus blocks the invasion of urothelial cells and avoids the requirement for antibiotic, reducing the risk of resistance [45] Antiadhesive FimH antagonists provide a therapeutic opportunity to prevent UTIs because they result in selection pressure on UPEC. FimH is therefore a suitable therapeutic target Most of the information regarding the mechanism (crystal structures) are coming from evidence on mannosides. However, crystal evidence demonstrates that the binding pocket is identical [3, 12, 46–48] As far as D-mannose, it was reported to bind FimH with a KD value of 2.3 uM [44] Furthermore, Scharenberg [49] compared different FimH antagonists (including D-mannose), belonging to different compound families and their affinities for FimH and eight human mannose receptors. D-mannose showed inhibition of binding for all proteins tested, including FimH, at a concentration of 50 mM (more than 90% inhibition). Results demonstrated that affinity between carbohydrates and pathogen is predominantly caused by the combined strength of multiple interactions with ligands: multivalent carbohydrates on the host cell and multimeric and/or clustered receptors on pathogens greatly support binding between the interaction partners [49]	7	8
N#8	D-mannose-binding occurs via reversible hydrophobic/hydrophilic interactions not altering the protein conformation Evidence confirmed binding of D-mannose. A function of the hydrophobic edge around the binding pocket of FimH may be to direct the sugar into the pocket in a manner that facilitates polar interactions [4, 33, 50] More specifically, the mannose ring makes 10 direct hydrogen-bonds to the side-chains of residues Asp54, Gln133, Asn135 and Asp140, and to the main chain of Phe1 and Asp47, and indirect water-mediated hydrogen-bonds via O2 to the side-chain of Glu133 and to the main chain oxygen of Phe1 and Gly14 [44]. The alpha-anomeric hydroxyl group O1 of mannose is involved in a hydrogen-bonding water network with the Asn138 and Asp140 side-chains, through a water molecule [44] Crystallographic studies showed that all hydroxyl groups of the D-mannose sugar ring bind in a negatively charged pocket of FimH making ten direct hydrogen-bonds with residues in the carbohydrate recognition domain (CRD) [43] Moreover, Tyr48 and Tyr137 residues are positioned so as to form a “tyrosine gate” to which dimannoside/oligomannosides form van der Waals interactions [35, 43, 44]. These interactions do not change the conformation of FimH or surface bacterial structures [4, 33] The lack of downstream effects or changes in FimH is demonstrated by crystallographic evidence demonstrating that the binding of D-mannose to FimH does not alter the protein conformation [44] Blocking the adhesion of FimH to cell receptors impedes the following invasion process and facilitates the bacterial removal with the urinary flux	5	7
N#9	The interaction between D-mannose and the bacterial FimH delineates a classical drug-receptor interaction, and thus a pharmacological action Evidence shows that the contact between D-mannose and FimH is due to specific interactions between specific atoms of the D-mannose and specific amino acids residues in a specific binding region of the adhesin [35, 50]. On the other side, such an interaction does not result in conformational changes of the bacterial protein leading to activation of intracellular pathways important for the intended effect, i.e., there is no activation of a signalling pathway which is a condition for a pharmacological effect of the receptor-ligand interaction [44]	–	3
N#10	D-mannose does not achieve its effect on pathogen adhesion by antibiotic-like bactericidal or bacteriostatic activity In vitro tests show that, once D-mannose is removed from the urine, or once exogenous D-mannose is washed away from the culture broth, the bacterium regains its full ability to grapple to epithelial cells. This fact clearly indicates that D-mannose does not impair vitality of the microbe, and therefore is not a biocide [45]. No bacteriostatic or bactericidal effects were observed in the strains tested in Marcon [34] In addition, it is well demonstrated that, unlike antibiotics and antiseptics such as nitrofurantoin, D-mannose does not induce resistance [12], also after long-term use [33] No alteration of FimH could be observed in vitro after removal of the D-mannose, nor in the D-mannose-treated bladder cells [33] D-mannose just prevents the bacterial binding to mannosylated eukaryotic receptors. In this sense, the 2012 EU Court stating that chlorhexidine 0.12% shall not be considered as a cosmetic [23], since the mechanism of action of chlorhexidine is pharmacological because killing the bacteria, is here not applicable	7	7
N#11	D-mannose shows a concentration-dependent effect According to the MDC 2022–5 guidelines [7], although not an exhaustive criterion, the presence of a dose–response correlation is indicative of a pharmacological, metabolic or immunological mode of action D-mannose action is dose-dependent [4, 51]. The IC50 for the anti-adhesive efficacy and anti-invasion activity of D-mannose were 0.51 mg/mL and 0.30 mg/mL, respectively, both with concentration-dependent inhibition [52] D-Mannose efficiently blocked the adhesive properties of all type 1 fimbriae-positive isolates in low concentration (0.2%, 2 mg/mL) [34]. Evidence demonstrated that no differences in bacterial growth were observed for D-mannose concentrations up to 10% (10 mg/mL) [33]. Besides, the effect of a high dosage (1.5%) of D-mannose on human epithelial cells was also evaluated and no macroscopic differences in the bladder epithelia cells were assessed (shape, integrity, adhesiveness, cytoplasmic vacuolization, proliferation, or cytotoxic effects) [33]	6	6
N#12	The interaction between FimH and D-mannose does not result in an immunological answer with regard to the main effect The specific effect of D-mannose is not directly connected to a specific interaction with antibodies, immunocompetent cells or other components of the human immune system but occurs in the bladder once D-mannose is excreted into the urine. There D-mannose acts creating a chemical barrier on FimH which prevents the adhesion, i.e., chemically impeding the FimH [2, 33]. D-mannose has positive immunoregulatory effects on T-cells in mice with autoimmune diabetes and airway inflammation, which however are not relevant for its effects to prevent urinary tract infections [53]	6	6
N#13	The intended effect of D-mannose does not involve metabolic responses downstream its interaction with FimH D-mannose prevents interaction between the body’s urothelial cells and UPEC by binding to bacterial FimH. The binding between FimH and the D-mannose does not trigger downstream metabolic processes in the bacterium and the host cell. The lack of downstream effects is demonstrated by the full reversion of the binding activity of *E. coli* in the agglutination assay, since FimH remains unmodified [37] Moreover, in addition to its binding inhibitory activity, D-mannose is scarcely used to sustain bacterial growth. Scribano et al. 2020 demonstrated that, in D-glucose deficiency, a second hierarchy in bacterial growth rates was shown encompassing D-fructose/L-arabinose followed by D-mannose [33] As a matter of fact, during infection *E. coli* has sufficient glucose in the bladder to sustain its metabolism and, thus, the high administered doses of D-mannose for prevention of UTIs have no effects on bacterial metabolism and growth [33] Besides, D-mannose does not interfere with the antibiotic activity and does not change bacterial morphology or motility compared to untreated bacterial cells [33] Analysis of the therapeutic efficacy of various phytotherapeutics and their antimicrobial compounds demonstrated that D-mannose showed no bacteriostatic effect at 10% (100 mg/mL) while efficiently blocking the adhesive properties of all type 1 fimbriae-positive isolates at much lower concentration (0.2%, 2 mg/mL) and showed no bacteriostatic effect [34] Evidence also shows that removal of D-mannose from FimH by applying mechanical forces left the adhesin fully proficient to bind to human urothelial mannosylated receptors [33]. Scribano et al. demonstrated that the clinical regimen of D-mannose to prevent acute UTIs (3 g/day for three days, then 1.5 g/day for 10 days) does not lead to FimH mutations that modify bacterial adhesiveness [33] No macroscopic differences in the bacterial shape, integrity, adhesiveness, cytoplasmic vacuolization, proliferation, or cytotoxic were observed in vitro with D-mannose [33] As already reported, D-mannose interaction with the FimH adhesin neither causes nor blocks signal transduction and subsequent biochemical reactions	7	7

The two-domain structures of FimH allows the type 1 pilus to bind by a catch-bond mechanism. The catch-bond in FimH is biphasic: under moderate force (such as during urination) FimH binds to ligands with higher affinity. In reality, the application of moderate mechanical flow force prompts the dissociation of the FimHLD and FimHPL subunits, resulting in a switch of the lectin domain from a state of low affinity to one of high affinity, up to 1000-fold greater.

FimH’s comparatively feeble affinity in static conditions favors the invasion of UPEC along the urinary tract, while its high affinity under moderate flow conditions (i.e., during urination) allows UPEC to be retained in the urinary tract. This phenomenon can be attributed to the dynamic interplay between flow-induced mechanical forces and the molecular structure of FimH.

The panel concluded that, on the basis of the definitions, the mechanism of D-mannose does not involve a metabolic or immunological action while there is an uncertainty regarding the possibility of a pharmacological action.

As a matter of fact, D-mannose interacts with a cellular component present in the user’s body (the bacterial adhesin) and prevents a pathological process (i.e. the bacterial adhesion and infection). Regardless of whether D-mannose activates an intracellular pathway in bacteria or not, its mechanism of action involves preventing bacterial binding to uroepithelial cells. As a result, the bacteria are subsequently eliminated through urine without any interaction with human tissue. From this prevention effect derives the well-established prevention clinical effect [54–57].

Uncertainty regards the features of the interaction between D-mannose and the bacterial adhesin and if they delineate a classical ligand-receptor interaction. On one side, the interaction is due to multiple bonds between specific atoms of D-mannose and specific aminoacids residues in a specific binding region of the adhesin. On the other side, such an interaction does not result in specific changes of the conformation of the bacterial protein and there is no activation of intracellular pathways important for the intended effect. From a regulatory perspective, the implications of this result, based initial assumptions, drives to the possible classification of alpha-D-mannose as a medical device.

Box 2 Adhesion of pathogenic organisms to host tissues is the required step to initiate the majority of infectious diseases. It is mediated by lectins present on the surface of the infectious organism that binds to complementary carbohydrates on the surface of the host tissues. Bacterial lectins typically take the form of elongated, submicroscopic multi-subunit protein appendages referred to as fimbriae (or pili).

In the case of uropathogenic E. coli (UPEC), the primary mechanism of disease involves the adherence of these bacteria to mannosylated proteins on the bladder epithelium, specifically uroplakins (UPIa). The FimH adhesin, located at the tip of the type I fimbria of E. coli, facilitates this interaction, making it a crucial virulence factor in UTI pathogenesis [46].

Adherence of P-fimbriated *E. coli* or of the isolated P fimbriae (also known as pyelonephritis-associated pili) to uroepithelial cells induces a two-way flow of biological crosstalk via the lectin bridge, affecting both partners. After adherence occurs, the target cells become activated, resulting in the production of cytokines that elicit acute inflammation and other disease symptoms. Meanwhile, in bacteria, the interaction triggers the up-regulation of signal transduction systems that enable responses to changing environments. The FimH subunits of *E. coli* serve not only to mediate bacterial adhesion but also to facilitate invasion of human bladder epithelial cells.

It is noteworthy that more than 90% of *E. coli* and other enteric bacteria express the type 1 fimbrial adhesin FimH, which is a lectin-like protein that specifically binds to terminal mannose or oligomannose residues on glycoproteins found in a wide range of tissues. Without adhesion to mannosylated proteins on UPIa, UPEC would remain free in the urine and be removed from the bladder during urination, preventing the initial UPEC infection from progressing into a symptomatic UTI.

**Figure 4:**
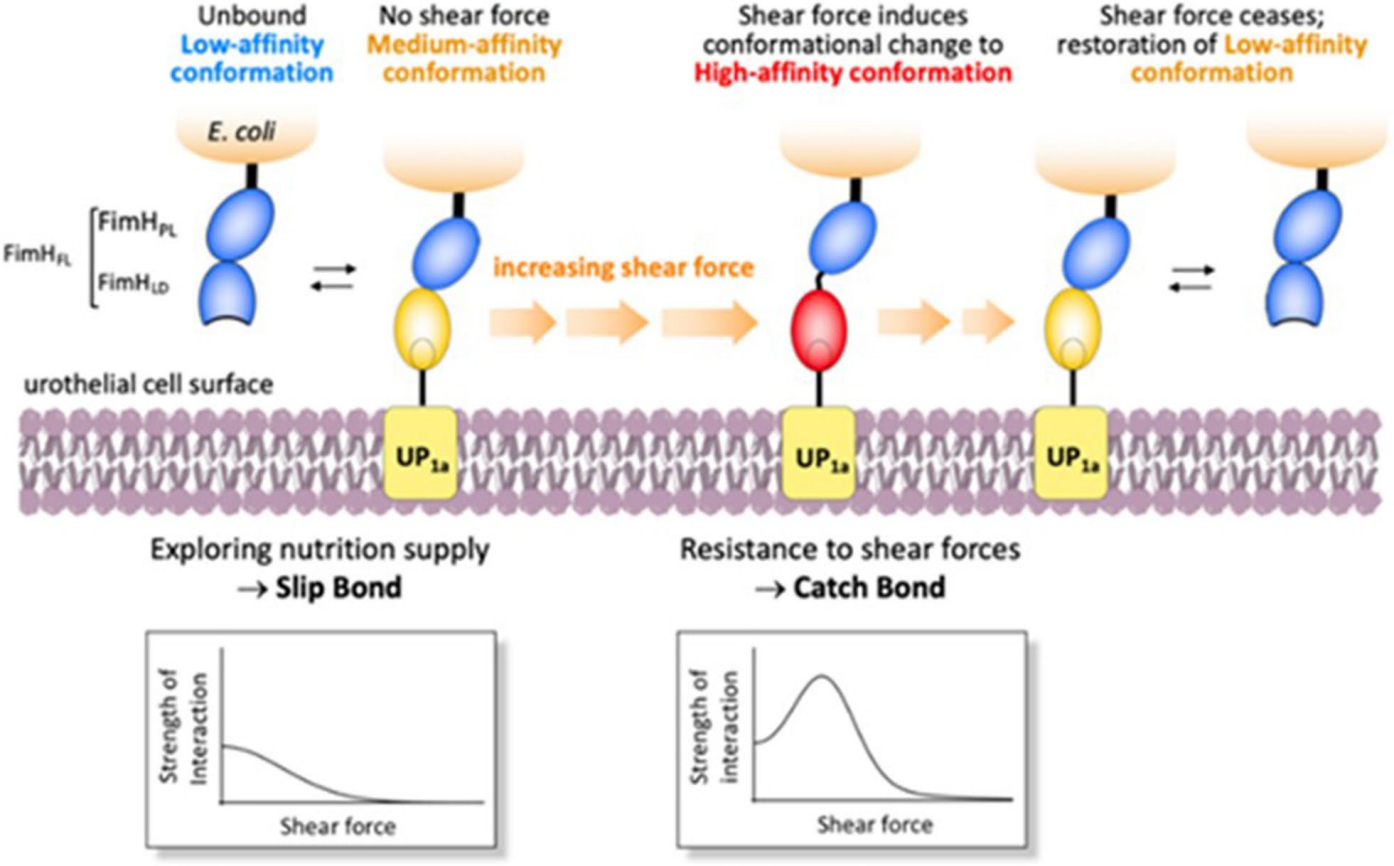
“Catch-bond” Mechanism for the Shear Force-Dependent Binding of FimH to Mannosylated Urothelial Surface. From Eris [48], Permission Requested.

To bind to terminal mannose units UPEC produces multiple 3 μm-long rod-like structures on their surface known as type 1 pili, constituted by Fim A, FimF and FimG and indeed FimH (Fig. 4) [38].

FimH is comprised two domains. The first is a C terminal pilin domain (FimHPD), which attaches FimH to the pilus rod through the neighbouring subunit FimG. The second FimH domain is the N-terminal lectin domain (FimHLD), which contains a mannose-binding pocket that can bind to mannoside/ oligomannoside sugars on the urothelial surface, mediating adhesion of UPEC to the urinary tract.

## Discussion

From a regulatory perspective, the fact that the pharmacological action is uncertain (as for the panel votes), drives toward the classification of D-mannose as a medical device. An interaction between the product and the bacteria within the body occurs, it is reversible and dose-dependent, but its nature is inert because it does not induce a direct response activating or inhibiting body processes or restoring, correcting, or modifying the physiological functions in the human being. Moreover, it must be considered that the action of D-mannose takes place, even if inside the bladder, outside the epithelium on bacteria that have not yet invaded the urothelial tissue. Hence, the modus operandi of this substance is not aimed towards the host’s anatomical components, but rather towards extrinsic structures, specifically bacteria, that exist outside of the host’s tissues.

Indeed, according to the EU jurisprudence [58, 59], products inducing a physiological effect cannot be automatically classified as medicinal products “by function” if the pharmacological, immunological, or metabolic effect is not demonstrated based on established scientific knowledge. Evidence does not demonstrate alpha-D-mannose pharmacological, immunological or metabolic action. Furthermore, the MEDDEV 2.1/3 rev.3 [24] and the updated version MDCG 2022–5 [7] define the pharmacological mode of action including two sequential steps: the interaction and the signal transduction pathway. The interaction by itself is not sufficient to determine the therapeutic effect [8].

The topic of borderline products is presently a subject of lively and ongoing debate within the scientific community, reflecting the nuanced complexities of this area of inquiry. The MDCG 2022–5 guidelines are the current instrument used to discuss the borderline nature of products. Using them in this research orientated the discussion toward a conservative hypothesis on the nature of the interaction between alpha-D-mannose and *E. coli*, being the guidelines the unique official document which introduces a specific definition of pharmacological effect. However, the guidelines do not have legal value and debate is ongoing in the scientific community around their completeness in the definitions provided. Nevertheless, regulatory considerations remain valid because guidelines include the MDR medical device definition.

Furthermore, Art. 2 related to medicinal products, included in the directive 2001/83/CE of the EU Parliament dated 6 November 2001- modified by the rule dated 31 March 2004, 2004/27/CE – does not apply to products whose functional quality of medicinal is not scientifically proven, although it cannot be excluded [21, 60]. In other words, in presence of scientific evidence providing a complete demonstration of alfa-D-mannose as a medicinal product, it remains possible its classification as a medical device.

A very recent European Court sentence [61] Judgment of the 7th Court Directive 93/42/EEC 19th January 2023, declared that “*when the main mode of action of a product have not been scientifically establish, this product cannot meet either the definition of the notion of medical device..nor to that of medicine by function*…” Taking into account the above, this could also be applied to alfa-D-mannose. As a consequence, as in the previous red yeast rice [58], “*the national courts must assess “on a case-by-case basis, whether the conditions relating to the definition of the concept of “medicinal product by presentation*” within the meaning of the Directive 2001/83 and amendments are satisfied.”

## Conclusions

The regulatory framework is constantly changing. New regulations and legal judgments can add further consideration. A request pending at the European Court will answer to the specific case of a medicinal product with insufficient evidence of the drug and its regulatory classification. The judgment of the Court (Fourth Chamber) 3 October 2013, reports the fact that each Member State can have a different regulatory classification for the same product (medicinal product or medical device), demonstrating that there is not a unique opinion on classifications but, mostly, that it is not even required [62].

Ultimately, it is unlikely that additional chemical evidence will emerge regarding the interaction between E. coli and natural products such as alpha-D-mannose, given the low affinity of the interaction. Research efforts are instead focused on synthetic mannosides and their potential for preventing urinary tract infections.

### Supplementary Information

Below is the link to the electronic supplementary material.

Supplementary file1 (DOCX 39 KB)

## Data Availability

Any further data that the reader light want to see is available upon request.

## Footnotes

1 Article 2, MDR 2017/745: For the purposes of this Regulation, the following definitions apply: [1] ‘medical device’ means any instrument, apparatus, appliance, software, implant, reagent, material or other article intended by the manufacturer to be used, alone or in combination, for human beings for one or more of the following specific medical purposes: — diagnosis, prevention, monitoring, prediction, prognosis, treatment or alleviation of disease, — diagnosis, monitoring, treatment, alleviation of, or compensation for, an injury or disability, — investigation, replacement or modification of the anatomy or of a physiological or pathological process or state, — providing information by means of in vitro examination of specimens derived from the human body, including organ, blood and tissue donations, and which does not achieve its principal intended action by pharmacological, immunological or metabolic means, in or on the human body, but which may be assisted in its function by such means”.
