# Supplementary material for: Nature of the Interaction of Alpha-D-Mannose and Escherichia coli Bacteria, and Implications for its Regulatory Classification. A Delphi Panel European Consensus Based on Chemistry and Legal Evidence
Source: Ther Innov Regul Sci. 2023 Aug 14;57(6):1153–66. doi: 10.1007/s43441-023-00548-8 (PMC10579141; doi:10.1007/s43441-023-00548-8)
Supplement: Supplementary file 1 — Supplementary file1 (DOCX 39 KB) [file 43441_2023_548_MOESM1_ESM.docx]

**Table S1: Reference studies: summary of findings**

| **Reference** | **Study type (in vivo, in vitro)** | **Main results** | **Implication for the research topic** |  |
| --- | --- | --- | --- | --- |
| Fein 1981 | In vitro | Strains from patients with  UTI agglutinated the yeast cells. Only 51% of 51 fresh isolates had mannose-sensitive adhesin activity. | Various strains belonging to several genera exhibited positive agglutinating activity. The agglutination reactions were generally strong and completely inhibited by 0.7% D-mannose. |  |
| Ofek 1981 | In vitro | *E. coli* excreted in only 2 of 24 urine specimens exhibited mannose-specific adherence. However, about half of the broth cultures from these specimens did so. | Mannose-specific adherence is indicative of the presence on the bacterial surface of adhesins  (lectins) binding the organisms to mannose residues on both epithelial and phagocytic cells. |  |
| Sharon 2006 | Review | In the urinary tract, the fimbriae mediate the binding of the bacteria to uroplakins Ia and Ib (two major glycoproteins of urothelial apical plaques). Anchorage of *E. coli* to the urothelial surface via fimbriae–uroplakin I interactions may play a role in bladder colonization. | According to crystallography investigations, eight  residues combine with mannose through hydrogen bonds and hydrophobic interactions. |  |
| Reid 1984 | In vitro | High incidence of a mannose-sensitive adhesin and a mannose-resistant/eluting adhesin among *E. coli* urinary isolates. | Microscopy confirmed fimbrial presence on 67% of strains producing a mannose resistant adhesin. |  |
| Hultgren 1986 | In vitro | Results indicate that type 1 pili are a predominant adhesin of UPEC | | |
| Hacker 1986 | In vitro | 73% of the UTI strains and 52% of the fecal isolates showed P-receptor  specificity; 17 % of the uropathogenic bacteria and 34 % of the fecal strains exhibited S, M or X-fimbriae antigens. | A high percentage of  P-fimbriated strains showed mannose-sensitive hemagglutination and hemolysin production. |  |
| Jones 1985 | In vitro | Type 1 pili are heteropolymeric mannose binding fibers produced by all members of the Enterobacteriaceae family. | The bipartite nature of the type 1 pilus was demonstrated |  |
| Ronald 2008 | In vitro: static and dynamic adhesion to mannose-coated surfaces of bacteria with different naturally occurring mutations in the Signal Peptides (SP) | SP-mediated membrane transport confers an adhesive phenotype to target cells or surfaces. SP mutations:   - decrease the number but increase the length of surface-expressed fimbriae - result in decreased attachment under flow - do not dramatically affect binding to bladder epithelial cells but decrease killing by neutrophils. | Under various levels of shear stress SP mutants adhered significantly less than wild type. Under static conditions bacterial binding to mannose was not affected by SP mutations, likely because the number of FimH-mannose bonds formed was high enough in all strains to resist post-incubation bacterial removal by washing. |  |
| Chen 2009 | In vivo: Construction of Chromosomal Mutant fimH Strains and in vivo fitness in mouse models | Epithelial invasion without Intracellular Bacterial Communities (IBC) formation also leads to bacterial clearance, which may be related to the ability of the bladder epithelial cells to expel UPEC. Involvement of FimH in any of several key UPEC infection processes (innate immune evasion, cytoplasmic replication, escaping expulsion, modulation of epithelial apoptosis and exfoliation) could disrupt IBC formation and explain the dichotomy between mannose binding and in vivo fitness seen with specific mutations. | FimH binding to mannose is necessary but not sufficient for UPEC to cause UTI. Previously unappreciated structure-function relationships in FimH direct an activity downstream of mannose binding that is necessary for IBC formation. |  |
| Hung 2002 | In vitro | The crystallographic structure of the FimH adhesin bound to its mannose receptor was determined. The binding pocket was characterized by site-directed mutagenesis and functional analysis. Elucidation of the molecular basis of a highly conserved host-pathogen interaction is necessary for the establishment of persistent UTI. The function of the hydrophobic ridge around the pocket may be to direct the sugar into the pocket by facilitating polar interactions within the FimH pocket. | The structure/function analysis demonstrated that the binding of the monosaccharide alpha-D-mannose is the primary bladder cell receptor for UOEC. This event requires a highly conserved FimH binding pocket. |  |
| Cusumano 2011 | In vivo | Small-molecular weight compounds (mannosides) inhibiting the FimH type 1 pilus lectin of UPEC optimized for oral bioavailability. | 95% of mannosides excreted in the urine unchanged and no toxicity was observed up to a dose of 200 mg/kg. |  |
| Scribano 2020 | In vitro | The low metabolic/energetic advantages for bacterial growth, the lack of selection of altered FimH adhesins after long-term D-mannose exposure, and the bladder cell tolerance emphasize the safe use of D-mannose in the treatment and prevention of UTIs caused by UPEC. | D-mannose masks the bacterial adhesin FimH, thereby preventing its binding to urothelial cells. High D-mannose concentrations have no effect on bacterial growth, neither bacterial viability, shape, or motility nor interfered with the activity of the tested antibiotics. Small amounts of glucose are normally present in urine from healthy subjects, so the dosages of D-mannose used in clinical practice are irrelevant for *E. coli* metabolism and growth. |  |
| Mydock-Mcgrane 2015 | In vitro | X-ray crystallographic binding studies of D-mannose  and early FimH antagonists demonstrated interactions | Biaryl (two-ring) mannosides are the largest and potentially useful tested class of FimH antagonists |  |
| Marcon 2019 | In vitro | D-mannose blocked the adhesion of all type 1 fimbriae-positive isolates at low concentration (0.2%). No bacteriostatic effect was observed. | | |
| Ribic 2019 | In vitro | Polyphenol mannosides (structurally similar to biaryl inhibitors) can act as high-affinity FimH ligands. | Crystal structure of FimH elucidated that binding site  of FimH is included in a polar mannose-binding pocket and a hydrophobic region surrounding the polar binding pocket. |  |
| Scharenberg 2012 | In vitro | The selectivity range of five FimH antagonists belonging to different compound families by comparing their affinities for FimH and eight human mannose receptors was investigated. No adverse side effects resulting from nonselective binding to the human receptors have to be expected. | Adverse side effects resulting from nonselective binding of monovalent FimH antagonists to the investigated mannose-binding lectins should not be considered a critical issue. The 10^5^-fold lower affinity for the tested human receptors as compared to the bacterial FimH lectin confirms a high selectivity safety range. Given the importance of multivalent ligand presentation in nature, monovalent α-D-mannopyranosides could be considered to exhibit only low affinities to human mannose receptors. |  |
| Pang 2012 | In vitro: structure–activity  and structure–property study on a series of biphenyl alpha-D-mannosides | The docking mode of alpha-D-mannosides was studied through different byphenyl derivatives. The authors investigated the structure–affinity relationship for specific ortho substituents on ring A of the biphenyl aglycone of the FimH antagonists. The correlation between van der Waals volumes of these substituents and the enthalpy term clearly indicates the importance of shape complementary. | Carbohydrate recognition domain of FimH with oligomannosides on urothelial cells can switch from an in-docking mode to an outdocking mode, depending on the structure of the antagonist. |  |
| Kalas 2017 | In vitro, in vivo | Positively selected residues allosterically modulate the equilibrium between two conformational states of FimH each of which involves mannose through distinct binding orientations. | | |
| Bhomkar 2010 | In vitro | Comparing the transcriptional responses of attached and unattached fimbriated cells, the profile of selected genes was similar whether wild-type FimH was bound to a mannose ligand or modified FimH was bound to a Ni2+‑NTA ligand. Identified genes were altered in response to fimbrial binding and were independent of the particular immobilized substrate.  The observed transcriptional response was a result of attachment of cells to the beads and was not due to their detachment.  A temporal analysis of cells binding  to mannose-functionalized surfaces revealed that the transcriptional response was magnified upon prolonged adhesion. | The transcriptomic response of *E. coli* upon attachment to the Ni+2-NTA substrate via the genetically modified FimH is similar to the transcriptional response observed in *E. coli* attached to mannose-agarose substrate via wild-type FimH. |  |
| Eris 2016 | In vitro | The mannose binding of four FimH variants with mannose-based ligands under static and hydrodynamic conditions demonstrated conformational variability of FimH. Additionally, the binding phenotype and the conformational equilibrium influenced the inhibitory  potencies of FimH variants. | It is important evaluating a FimH  ligand with respect to the conformational variability of the  FimH adhesin. |  |
| Hutton 2020 | In vitro, review | A report of progress in the development of FimH targeting mannose-based analogues with potential applications against UPEC-induced UTIs. Three categories of mannose-based FimH inhibitors: oligosaccharide-based FimH inhibitors, α-D-mannopyranoside-based inhibitors and polyvalent mannose-based inhibitors. Mannose-capped oligosaccharides most accurately reflect the structure of natural FimH ligands, yet oral bioavailability and complex chemical synthesis are still issues. The relatively weaker affinity of FimH under static conditions favours invasion of UPEC along the urinary tract during static conditions, while the high affinity of FimH under moderate flow conditions enables UPEC to be retained in the urinary tract during urination. By synthetic α-linked mono- and D-mannosides, used to represent the natural terminal α-db-mannoside moieties present on FimH targeted glycoproteins in the bladder, kinetic and structural characterization of the binding properties of FimH under both static and flow conditions were evaluated. The increased affinity of FimH under flow conditions compared to static conditions was ligand independent. | D-mannosides bound with higher affinity compared to monosaccharides, with the difference in affinity determined by the rate of spontaneous ligand dissociation. Under static conditions FimH binds to all-natural terminal α-D-mannoside with medium affinity, while under flow conditions FimH binds to all D-mannosides at a 2000-fold higher affinity with a 70,000-fold decrease in ligand dissociation rate and 30- fold increase in ligand association rate. |  |
| Bouchaert 2005 | In vitro | Crystallographic evidence shows that the lectin domain of FimH is a stable and functional entity. Alkyl mannosides have nanomolar affinities for FimH. | Relative FimH affinities correlate exceptionally well with the relative concentrations of the same glycans needed for the inhibition of adherence of type 1 piliated *E. coli*. |  |
| Sager 2017 | Review | The bacterial lectin FimH is situated in a deep mannose-binding site, which, together with both a catch-bond mechanism and multivalency, improves the affinity of the carbohydrate–FimH interaction. The latter can be further improved. | | |
| Madison 1994 | In vitro | The differences in sugar-binding specificity between *E. coli* and *K. pneumonia* FimH fimbrial subunits is due to the fimbrial shafts carrying the adhesin molecules in a functionally competent form at the distal tips. | | |
| Feenstra 2017 | In vitro | Comparison of adhesion to endothelium under static and shear stress conditions revealed that E50 and T53 were essential under shear stress. This only partly correlated with binding to mannosylated substrates under identical assay conditions, demonstrating that there are important differences for the adhesion of isolated mannosylated substrates and mammalian cells. | The importance of the mannose binding pocket in critically influencing the adhesion under flow was identified for the first time. |  |
| Scaglione 2021 | Review | From both a scientific and a regulatory point of view, a pharmacological action can be established based on three criteria: 1) the existence of an interaction molecule-cellular constituent of  an organism, 2) such interaction induces a direct response, activating or inhibiting normal processes in the organism, and 3) the induced response should restore, correct, or modify the physiological functions in the person.  According to the EU jurisprudence, products containing a substance which has a physiological effect cannot be automatically classified as medicinal products “by function” if the pharmacological,  immunological, or metabolic effect is not demonstrated based on the established scientific knowledge | The interaction between D-mannose and FimH adhesin does not produce any activation or inhibition response either to the bacteria or to the urothelium epithelial cells. D-mannose inhibition of bacteria adhesion seems based on inert physical interactions (comparable to those between the platelets and calcium alginate or oxidized cellulose). |  |
| Michaels 1983 | In vivo | D-mannose significantly reduces bacteriuria within 1 day; their efficacy depends upon the concentration of both saccharide and bacteria. | | |
| Pani 2022 | In vivo | D-mannose inhibits the adhesion of *E. coli* to urothelial cells at  high concentrations, whereas inhibition of invasion occurred at much lower concentrations. | | |
| Ala-Jakkoola 2022 | Review | D-mannose has positive immunoregulatory effects on T-cells in mice with autoimmune diabetes and airway inflammation.  The role of regulatory T-cells, UTI and D-mannose needs to be better explored. The affinity FimH-mannosides shown in vitro and animal models will presumably prevent bacterial entry and infection of urinary tract cells. | | |
| Zhang 2017 | In vitro; Treg cell differentiation in human and mouse cells | Supraphysiological levels of D-mannose suppresses immunopathology in mouse models of autoimmune diabetes and airway inflammation, and increases the proportion of Foxp3+ regulatory T cells in mice. In vitro, D-mannose stimulates Treg cell differentiation in human and mouse cells. | D-mannose induces the generation of Treg cells from naive CD4+ T cells by enhancing TGF-β signaling. Integrin αvβ8 and ROS pathways were required for Treg cell induction by D-mannose in T cells. Molecular studies showed that D-mannose increased integrin α_v_β_8_expression, FAO, and ROS levels in T cells. |  |
| Pak 2001 | In vitro | Tamm-Horsfall Protein (THP) the glycoprotein in mammalian urine, may serve as a soluble receptor for type 1 fimbriated *E. coli*, coating the adhesins and preventing their binding to urothelial receptors, thereby facilitating their elimination from the urinary tract. THP contains high-mannose residues capable of interacting with type 1 fimbriated *E. coli*. | THP binds specifically to type 1 fimbriated *E. coli.* The binding is highly specific and saturable and can be inhibited by D-mannose and abolished by endoglycosidase H treatment of THP, suggesting that the binding is mediated by the high-mannose  moieties of THP. |  |
| Schwartz 2013 | In vitro  In vivo (mice) | FimH can adopt a compact conformation when incorporated into the pilus. FimH sequence modulates IBC number and modifies ability to persist during chronic cystitis. FimH’s ability to switch between conformations is important in pathogenesis. Positively selected residues modulate fitness during UTI by affecting FimH conformation and function. | Positively selected residues outside of the FimH mannose-binding pocket affect transitions between low and high-affinity FimH conformations.  The FimH of the two prototypical UPEC isolates shows high affinity for mannose when complexed with FimC and an intermediate affinity when complexed with FimG in a tip-like FimChisGH complex. |  |
| Zhou 2001 | In vivo | In mouse, urothelial plaques uroplakin Ia is the major bacterial binding protein and the only receptor for bacterial FimH | The structurally related uroplakins Ia and Ib are glycosylated differently: uroplakin Ia serves as the urothelial receptor for the type 1-fimbriated *E. coli.* |  |
